# Serum proteins mediate depression’s association with dementia

**DOI:** 10.1371/journal.pone.0175790

**Published:** 2017-06-08

**Authors:** Donald R. Royall, Safa Al-Rubaye, Ram Bishnoi, Raymond F. Palmer

**Affiliations:** 1 Department of Psychiatry, the University of Texas Health Science Center, San Antonio, Texas, United States of America; 2 Department of Medicine, the University of Texas Health Science Center, San Antonio, Texas, United States of America; 3 Department of Family and Community Medicine, the University of Texas Health Science Center, San Antonio, Texas, United States of America; 4 South Texas Veterans’ Health System Audie L. Murphy Division Geriatric Research Education and Clinical Care Center, San Antonio, Texas, United States of America; 5 Department of Psychiatry, the Medical College of Georgia, Augusta, Georgia, United States of America; Universiteit Antwerpen, BELGIUM

## Abstract

The latent variable “δ” (for “dementia”) uniquely explains dementia severity. Depressive symptoms are independent predictors of δ. We explored 115 serum proteins as potential causal mediators of the effect of depressive symptoms on δ in a large, ethnically diverse, longitudinal cohort. All models were adjusted for age, apolipoprotein E, education, ethnicity, gender, hemoglobin A1c, and homocysteine, and replicated in randomly selected 50% subsets. Alpha1-antitrypsin (A1AT), FAS, Heparin-binding EGF-like Growth Factor (HB-EGF), Insulin-like Growth Factor-1 (IGF-1), Luteinizing Hormone (LH), Macrophage Inflammatory Protein type 1 alpha (MIP-1α), Resitin, S100b, Tissue Inhibitor of Metalloproteinase type 1 (TIMP-1), and Vascular Cell Adhesion Molecule type 1 (VCAM-1) each were partial mediators of depression’s association with δ. These proteins may offer targets for the treatment of depression’s specific effect on dementia severity and Alzheimer’s Disease (AD) conversion risk.

## Introduction

Major Depression has been identified as a risk factor for incident clinical “Alzheimer’s disease (AD)” [1–2]. However, the mechanism(s) involved is not clear. Depressive symptoms may effect clinical dementia independently of neurodegeneration [3]. The subset of clinically diagnosed “AD” cases who lack significant Aβ deposition by positron emission tomography are more likely to be taking antidepressants [4–5]. On the other hand, the Serotonin Selective Reuptake Inhibitor (SSRI) citalopram has been shown to alter amyloid beta (Aβ) deposition and /or clearance [6].

Dementia cannot be diagnosed on the basis of cognitive impairment alone. Instead, cognitively mediated functional impairments are required [7]. We have discovered that Instrumental Activities of Daily Living (IADL) are related to cognitive performance largely through Spearman’s General Intelligence factor, “*g*” [8–9]. Thus, dementia may represent a disruption of intelligence, independently of any concurrent changes in domain-specific variance (e.g., Memory, Language, and /or “Executive Function”).

Cognition’s association with functional status is best assessed as a latent variable in a Structural Equation Model (SEM) framework. We have eschewed clinical diagnostic categories (e.g., “Mild Cognitive Impairment (MCI)” or “Major Neurocognitive Disorder”) in favor of one such latent “dementia phenotype”. δ (for “dementia”) is a latent variable. It embodies *g*’s association with IADL [10] and can be extracted from any cognitive battery that contains a measure of functional status.

δ can be “reified” as a composite “d-score”. d-scores achieve remarkable diagnostic accuracy relative to clinicians, are strongly associated with dementia severity cross-sectionally and longitudinally [11–12], and are free of cultural and linguistic bias [13–14]. The d-score also effectively converts dementia from a category to a dimension. We can rank order individuals, *even controls*, for their dementia severity. Each quintile increase in the d-score of non-demented persons increases their 5-year AD conversion risk by 50%. The risk of MCI cases increases almost threefold [15]. Because of δ’s strong and specific association with dementia (across diagnoses)[11], observed dementia severity becomes the sum of independent δ-related processes. Correcting any of them should improve dementia severity.

Age, gender, depressive symptoms and apolipoprotein E (APOE) ε4 allele burden are independent predictors of δ [16]. Their associations with clinical dementia and dementia conversion may thereby be constrained to biological processes that affect intelligence. We recently identified serum proteins mediators of Age’s [17] and ε4’s [18]-specific associations with δ.

In this analysis and by identical methods, we combine SEM with longitudinal data from the Texas Alzheimer’s Research and Care Consortium (TARCC) to explore more than 100 serum proteins as potential mediators of depressive symptoms’ specific associations with δ. Our model is constructed such that any significant mediator of the effect of depressive symptoms on prospective δ scores can be interpreted causally. Thus, they may offer targets for the remediation of depression-specific cognitive impairments.

## Materials and methods

### Subjects

Subjects included n = 3385 TARCC participants, including 1240 cases of Alzheimer’s Disease (AD), 688 “Mild Cognitive Impairment “(MCI) cases, and 1384 normal controls (NC). Each underwent serial annual standardized clinical examinations. Categorical clinical diagnoses of “AD”, “MCI” and “NC” were established through consensus. The diagnosis of AD was based on National Institute for Neurological Communicative Disorders and Stroke-Alzheimer’s Disease and Related Disorders Association (NINCDS-ADRDA) criteria.[19] The diagnosis of MCI was based on site-specific consensus-based clinical diagnoses derived from all available information but without reliance on specific neurocognitive tests and /or cut-scores. “All available information” included the results of TARCC’s entire neuropsychological battery, clinical evaluations, informant interviews, and any available outside medical records. We could not easily use cut-scores because MA norms are not available for many measures. Institutional Review Board approval was obtained at each site and written informed consent was obtained for all participants.

### δ’s indicators

δ was constructed from data acquired at Wave 2, i.e., one year after collection of the GDS and serum biomarkers. δ’s indicators included Logical Memory II (LMII)[20], Visual Reproduction I (VRI)[20], the Controlled Oral Word Association (COWA)[21], Digit Span Test (DST)[20] and Instrumental Activities of Daily Living (IADL)[22]. All tests were available in Spanish translation. The latent variables’ indicators were not adjusted for this analysis. The resulting unadjusted δ homolog was validated by its association with dementia severity, as measured by the Clinical Dementia Rating Scale sum of boxes (CDR)[23] and by Receiver Operating Curve (ROC) analysis.

Logical Memory II:[20] Following a thirty minute delay, the subject recalls two paragraphs read aloud.

Visual Reproduction I:[20] The subject immediately reproduces a set of figures after a brief exposure.

The Controlled Oral Word Association (COWA):[21] The patient is asked to name as many words as they can in one minute, beginning with a certain letter.

Digit Span Test (DST):[20] The DST sums the longest set of numbers the subject can immediately recall in correct order (forwards and backwards).

Instrumental Activities of Daily Living (IADL):[22] IADL’s were assessed using informant ratings. Functional abilities were rated on a Likert scale ranging from 0 (no impairment) to 3 (specific incapacity). A total IADL score calculated as the sum of all eight items.

### Covariates

All observed measures in the structural models were adjusted for age, APOE ε4 burden education, ethnicity, gender, homocysteine (HCY), and hemoglobin A1c (HgbA1c).

#### Clinical covariates

Age: Self-reported age was reconciled with date of birth and coded continuously.

Education: Education was coded continuously as years of formal education.

Ethnicity: Ethnicity was determined by self-report and coded dichotomously as “Hispanic” and “non-Hispanic”.

Gender: Gender was coded dichotomously.

#### Biological covariates

Measurements of HCY, HgbA1c and APOE ε4 genotyping were performed in the Ballantyne laboratory at the Baylor College of Medicine. HgbA1c was measured in whole blood by the turbidimetric inhibition immunoassay (TINIA). HCY was measured in serum using the recombinant enzymatic cycling assay (i.e., Roche Hitachi 911).

#### APOE genotyping

APOE genotyping was conducted using standard polymerase chain reaction (PCR) methods [23]. APOE ε4 burden was coded 0–2 based on the number of ε4 alleles.

### Clinical correlates

The Clinical Dementia Rating Scale sum of boxes (CDR-SB)[24]: The CDR is used to evaluate dementia severity. The information necessary to those ratings is collected during an interview with the patient and their caregiver. Each CDR domain is rated on a scale of 0.0–3.0. A total CDR-SB score is calculated as the sum of all six domains.

The Geriatric Depression Rating Scale (GDS) [25–26]: Depressive Symptoms were assessed at baseline (Wave 1). GDS scores range from zero-30. Higher scores are worse. Scores > 10/30 are considered to be clinically significant. The GDS is valid in demented persons. [26]

The Mini-Mental Status Exam (MMSE) [27]: The MMSE is a well known and widely used test for screening cognitive impairment. Scores range from 0 to 30.

### Biomarker mediators

TARCC’s methodology has been described elsewhere [28]. Serum samples were sent frozen to Rules-Based Medicine (RBM) in Austin, TX. There they were assayed without additional freeze-thaw cycles. RBM conducted multiplexed immunoassay via their human multi-analyte profile (human MAP). Serum samples were sent frozen to Rules-Based Medicine (RBM) in Austin, TX. There they were assayed without additional freeze-thaw cycles. RBM conducted multiplexed immunoassay via their human multi-analyte profile (human MAP). A complete listing of the biomarker panel we employed is available at http://www.rulesbasedmedicine.com.

We ran all RBM analyses in duplicate and discarded data when the duplicate values differed by > 5%. All values recorded by RBM as “LOW” were recorded and analyzed. If more than 50% of the samples for a given analyte were recorded as “LOW”, all readings for that analyte were dropped. If less than 50% of the analytes were recorded as “LOW”, the LOW values were recorded as the least detectable dose (LDD) divided by two. Raw biomarker data were inspected to ascertain their normality. Data points beyond 3.0 standard deviations (SD) about the mean were labeled as “outliers” and deleted. Logarithmic transformation was used to normalize highly skewed distributions. The data were then standardized to a mean of zero and unit variance. TARCC’s RBM biomarkers exhibit significant batch effects. Therefore, each biomarker was additionally adjusted for dichotomous dummy variables coding batch.

### Statistical analyses

#### Analysis sequence

This analysis was performed using Analysis of Moment Structures (AMOS) software. [29] The maximum likelihood estimator was chosen. All observed indicators were adjusted for age, education, ethnicity and gender. Co-variances between the residuals were estimated if they were significant and improved fit.

We used the ethnicity equivalent δ homolog (“dEQ”) as previously described. [30] That homolog has been reported to 1) exhibit factor equivalence across ethnicity, 2) have excellent fit (i.e., *χ*^2^/df = 181/24, p < 0.001; CFI = 0.97; RMSEA = 0.05), 3) have acceptable factor determinacy by Grice’s Method [31], 4) exhibit factor equivalence across ethnicity, 5) to be strongly correlated with dementia severity as measured by the CDR (r = 0.99, p <0.001) and 6) to exhibit an AUC of 0.97 (CI: 0.97–0.98) for the discrimination between AD cases and controls (in Wave 2 TARCC data). For the purposes of this analysis, dEQ was again constructed in Wave 2 data, but without any covariates, specifically age, ethnicity, GDS, gender, HCY, HGbA1c and APOE ε4 burden.

dEQ and g’ factor weights were applied to Wave 2 observed data to generate Wave 2 dEQ and g’ composite scores (i.e., dEQ w2 and g’ w2, respectively). g’ is dEQ’s residual in Spearman’s g. The composite scores were used as observed outcomes in models of a baseline APOE ε4 allele’s direct association with covariate adjusted Wave 2 dEQ.

**Fig 1:**
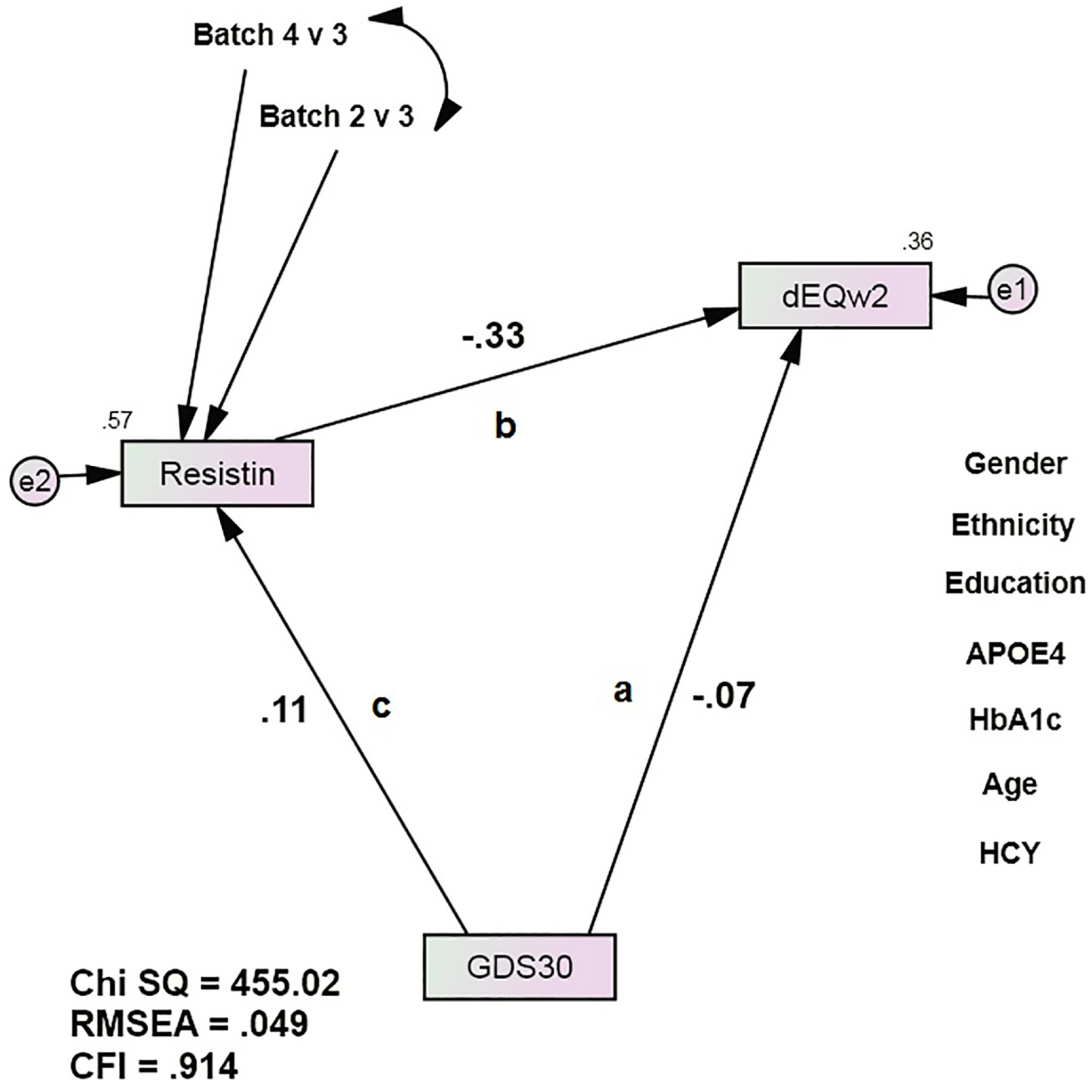
Resistin mediates the GDS30’s direct association with future dementia severity, as measured by dEQ. APOE = apolipoprotein e4 status; CFI = Comparative Fit Index; GDS = Geriatric Depression Scale; HCY = serum homocysteine; HgbA1c = serum hemoglobin A1c; IGF-BP2 = Insulin-like Growth Factor Binding Protein 2; RMSEA = Root Mean Square Error of Association. *All observed variables except GDS are adjusted for age, APOE, education, ethnicity, gender, HCY, and HgbA1c (paths not shown for clarity). The covariates are densely intercorrelated.

Next, we constructed a longitudinal mediation model in SEM (Fig 1). Such models can arguably be interpreted causally.[32] Path “a” represents the GDS’ direct association with Wave 2 dEQ scores. We modeled GDS as a continuous measure of depressive symptoms, not “depression” as a categorical diagnosis. Categorical diagnoses reduce information (relative to continuous measures) and introduce measurement bias (inherent to the application of diagnostic criteria). Morever, TARCC’s selection bias (as an “AD” study), selects against more impaired depressive cases.

Path “b” represents the biomarker’s independent effect on dEQ, measured at Wave 1. When both were significant, we considered path “c”. Bonferroni correction to p <0.001 was used to offset the potential for Type 2 error after multiple comparisons. The biomarker’s mediation effect on the APOE ε4 allele’s direct association can then be calculated by MaKinnon’s method [33].

The mediation models were constructed in a randomly selected subset of TARCC participants, comprising approximately 50% of the subjects (i.e., Group 1: n = 1691). As a test of each model’s generalizability to the remainder (n = 1694), each mediation path’s significant direct association was constrained across the two groups, and model fit compared across constrained and unconstrained conditions.[34–35] Mediation effects were calculated among Group 1 participants in the constrained models.

#### Missing data

We used the newest instance of TARCC’s dataset (circa 2016). The entire dataset was employed. Clinical diagnoses were available on 3385 subjects, 2861 of whom had complete data for δ’s cognitive indicators and covariates. Modern Missing Data Methods were automatically applied by the AMOS software.[36] AMOS employs Full information Maximum Likelihood (FIML). [37–38] Only the ROC analyses, performed in Statistical Package for the Social Sciences (SPSS) [39], were limited to complete cases.

#### Fit indices

Fit was assessed using four common test statistics: chi-square, the ratio of the chisquare to the degrees of freedom in the model (CMIN /DF), the comparative fit index (CFI), and the root mean square error of approximation (RMSEA). A non-significant chisquare signifies that the data are consistent with the model [40]. However, in large samples, this metric conflicts with other fit indices (insensitive to sample size) show that the model fits the data very well. A CMIN/DF ratio < 5.0 suggests an adequate fit to the data. [41] The CFI statistic compares the specified model with a null model. [42] CFI values range from 0 to 1.0. Values below 0.95 suggest model misspecification. Values approaching 1.0 indicate adequate to excellent fit. An RMSEA of 0.05 or less indicates a close fit to the data, with models below 0.05 considered “good” fit, and up to 0.08 as “acceptable“. [43] All fit statistics should be simultaneously considered when assessing the adequacy of the models to the data.

## Results

The demographic characteristics of TARCC’s sample are presented in Table 1. The mean GDS score was 5.60 ± 5.25, i.e., within the normal range. N = 537 (17.9%) scored ≥10 /30 (i.e., in the “depressed” range). The unadjusted wave 2 dEQ achieved a high AUC for the discrimination between AD cases and NC (AUC = 0.953; CI: 0.946–0.960). g”s AUC for the same discrimination was at a near chance level [AUC = 0.536 (CI: 0.514–0.558)]. This is consistent with past findings, across batteries, in this and other cohorts.

**Table 1:** Descriptive statistics.

Variable	N	Mean (SD)
**Age** (observed)	3381	70.88 (9.48)
**APOE e4 alleles** (1 = e4+, n = 1223)	3154	0.39 (0.49)
**CDR (Sum of Boxes)**	3306	2.42 (3.35)
**COWA**	3381	8.41 (3.49)
**DIS**	3381	8.89 (3.01)
**EDUC** (observed)	3381	13.24 (4.25)
**Ethnicity** (1 = MA, n = 1189)	3381	0.36 (0.47)
**GDS**_**30**_ (observed)	3005	5.60 (5.25)
**Gender** (♂ = 1, n = 1281)	3312	0.39 (0.49)
**IADL (Summed)**	3381	10.48 (4.52)
**MMSE**	3311	25.52 (4.76)
**WMS LM II**	3381	8.05 (4.30)
**WMS VR I**	3381	7.88 (3.68)
**Complete Cases**	2861	

CDR = Clinical Dementia Rating scale; COWA = Controlled Oral Word Association Test; DIS = Digit Span Test; GDS = Geriatric Depression Scale; IADL = Instrumental Activities of Daily Living; MMSE = = Mini-mental State Exam (Folstein, Folstein & McHugh, 1975); SD = standard deviation; WMS LM II = Weschler Memory Scale: Delayed Logical Memory; WMS VR I = Weschler Memory Scale: Immediate Visual Reproduction.

The Base Model fit well [*χ*^2^ = 84.42 (11), p <0.001; CFI = 0.978; RMSEA = 0.044]. Independently of the covariates (i.e., age, APOE ε4 burden, education, ethnicity, gender, HCY, and Hgb A1c), baseline GDS score was significantly directly associated with Wave 2 dEQ (r = -0.11, p<0.001), and weakly with the Wave 2 g’ composite (r = -0.03, p = 0.03). g’ was then dropped from consideration. The baseline GDS score’s significant association with Wave 2 dEQ scores was in a negative direction suggesting an adverse effect on future observed cognitive performance.

The mediation models all had acceptable fit [e.g., resistin: *χ*^2^ = 455.02 (17), p < 0.001; CFI = 0.914; RMSEA = 0.049 (Fig 1)]. alpha1-antitrypsin (A1AT), FAS, Heparin-binding EGF-like growth factor (HB-EGF), Insulin-like Growth Factor-1 (IGF-I), Luteinizing Hormone (LH), Macrophage Inflammatory Protein type 1 alpha (MIP-1a), Resitin, S100b, Tissue Inhibitor of Metalloproteinase type 1 (TIMP-1), and Vascular Cell Adhesion Molecule type 1 (VCAM-1) achieved statistically significant mediation effects after Bonferroni correction for multiple comparisons (Table 2). A1AT and resistin have previously been recognized depression-specific serum protein biomarkers. [42–43] All the identified proteins were partial mediators. Resistin’s effect was the largest (z = -4.54, p <0.01; 33.2%). We did not test multivariate mediations or interactions.

**Table 2:** Mediation effects (Class 1).

Mediating Biomarkers	Adjusted Path a (Fig 1)	Z (p)	Effect (%)
alpha1-antitrypsin (A1AT)*[44–45], ![18]	-0.14, p < 0.001	2.93 (0.002)	24.0
FAS![17–18]	-0.09, p < 0.001	-3.14 (<0.001)	20.1
Heparin-binding EGF-like growth factor (HB-EGF)![18]	-0.13, p < 0.001	3.16 (<0.001)	23.2
Insulin-like Growth Factor-1 (IGF-I)![17–18]	-0.09, p < 0.001	-5.78 (<0.001)	14.0
Luteinizing Hormone (LH)![18]	-0.08, p < 0.001	-5.16(<0.001)	18.7
Macrophage Inflammatory Protein type 1 alpha (MIP-1a)![18]	-0.13, p < 0.001	3.06 (0.001)	13.8
Resitin*[44–15], ![17–18]	-0.07, p < 0.001	-4.54 (<0.001)	33.2
S100b*[46], ![17–18]	-0.13, p < 0.001	3.38 (<0.001)	18.3
Tissue Inhibitor of Metalloproteinase type 1 (TIMP-1)![18]	-0.13, p < 0.001	3.23 (<0.001)	20.4
Vascular Cell Adhesion Molecule type 1 (VCAM-1)![18]	-0.12, p < 0.001	2.53 (0.006)	9.6

*Previously recognized depression-specific serum protein biomarker

^!^Previously recognized GDS adjusted biomarkers

Beta2-microglobulin (b2M), Chromogranin A, and Tumor Necrosis Factor receptor type II (TNF-RII) were associated with the GDS, but unrelated to dEQ by path b. b2M and TNF-RII have been previously recognized as depression-specific serum protein biomarkers. [44–45] However, although they might be related to cognitive performance (independently of δ), they cannot explain depression’s dementing aspects.

Table 3 presents the GDS-independent dEQ biomarkers. These include all but one (i.e., b2M) of those that we have previously associated with δ in GDS adjusted models. Also in this list are additional proteins [i.e., Apolipoprotein CIII (APOCIII), Brain-Derived Neurotrophic Factor (BDNF), cortisol, Epidermal Growth Factor (EGF), and Prolactin (PRL)] that have been previously associated with depression in other samples.[47–48] TARCC’s remaining protein biomarkers were related neither to the GDS, nor to dEQ.

**Table 3:** GDS-independent dEQ biomarkers (unrelated to the GDS by Path c).

Adiponectin (APN)![17]
Agouti-Related Protein (AgRP)![18]
alpha2-macroglobulin (α2M)‡[30, 49], ![18]
alpha-Fetoprotein (α-FP)![18]
Amphiregulin (AREG)![18]
angiopoetin-2N (ANG-2N)![17–18]
Angiotensin Converting Enzyme (ACE)![18]
Angiotensinogen![18]
apolipoprotein A1(APOA1)![18]
Apolipoprotein CIII (APOCIII)*[44–45], ![18]
AXL![18]
Betacellulin![18]
Bone Morphogenic Protein 6![18]
Brain-Derived Neurotrophic Factor (BDNF)*[44–45], ![18]
CD40![17–18]
Cancer Antigen 125 (CA 125)![18]
Cancer Antigen 19–9 (CA 19–9)![18]
Compliment 3 (C3)![17–18]
Connective Tissue Growth Factor (CTGF)![18]
Cortisol*[44–45], ![18]
C Reactive Protein (CRP)![18]
Creatinine Kinase-MB (CK-MB)![17–18]
Eotaxin-3![18]
Epidermal Growth Factor (EGF)*[44–45], ![18]
Epidermal Growth Factor Receptor 1 (EGFR)![17–18]
Epiregulin (EREG)![18]
Factor VII![18]
FAS-Ligand (FAS-L)![18]
Follicle stimulating hormone (FSH)![17–18]
Glutathione S-Transferase (GST)![17–18]
Granulocyte Colony Stimulating Factor (GCSF)![17–18]
Hepatocyte Growth Factor (HGF)![18]
Immunoglobulin A (IgA)
Immunoglobulin M (IgM)![17]
Insulin![17–18]
Insulin-like Growth Factor-Binding Protein 2 (IGF-BP2)![17–18]
Interferon-gamma‡[49], ![18]
Interleukin 1 receptor antagonist (IL-1ra)![18]
Interleukin 3 (IL-3)![18]
Interleukin 5 (IL-5)![17–18]
Interleukin 7 (IL-7)![18]
Interleukin 8 (IL-8)![17–18]
Interleukin 10 (IL-10)‡[49].! [18]
Interleukin 12-p40 (IL-12p40)‡[49], ![18]
Interleukin 13 (IL-13) ‡[30, 49], ![18]
Interleukin 15 (IL-15)†[50], ![18]
Interleukin 16 (IL-16)![18]
Lipoprotein a (LPA)![18]
Macrophage Inflammatory Protein type 1 beta (MIP-1b)![18]
Matrix Metalloproteinase type 3 (MMP-3)![18]
Monocyte Chemotactic Protein type 1 (MCP-1)![18]
Myoglobin (MyG)![17–18]
Pancreatic Polypeptide (PP)![17–18]
Plasminogen Activator Inhibitor type 1 (PAI-1)![17–18]
Platelet-Derived Growth Factor (PDGF)![17–18]
Progesterone![17–18]
Prolactin (PRL)†[63], *[44–45], ![18]
Prostate Specific Antigen (PSA)![18]
Pulmonary and Activation-Regulated Chemokine (PARC)![18]
Serum Amyloid P (SAP)![17–18]
Serum Glutamic Oxaloacetic Transaminase (SGOT)![18]
Soluable Advanced Glycosylation End Product-Specific Receptor) (sRAGE)![18]
Sortilin![18]
Stem Cell Factor (SCF)‡[49], ![18]
Tenascin C![17–18]
Testosterone![17–18]
Thrombopoietin (THPO)‡[30, 49], ![18]
Thrombospondin-1 (THBS1)![18]
Thyroxine Binding Globulin (TBG)![17–18]
Tissue Factor (TF)![18]
Tissue Growth Factor alpha (TGF-α)![18]
Tumor Necrosis Factor-Related Apoptosis-Inducing Ligand Receptor 3 (TRAIL-R3)![18]
Tumor Necrosis Factor alpha (TNF-α)‡[49], ![18]
Vitamin D Binding Protein (VDBP)†[51],![18]
Vascular Endothelial Growth Factor (VEGF)![18]
von Willebrand Factor (vWF)‡[30, 49], ![17–18]

* Previously recognized depression-specific serum protein biomarker

^‡^Previously recognized δ biomarkers in Non-Hispanic White TARCC participants only

^†^Previously recognized ethnicity adjusted δ biomarkers

^!^Previously recognized GDS adjusted biomarkers

## Discussion

We have surveyed more than 100 potential mediators of the GDS’s specific and significant association with δ. Our sample size was large, and we were powered to detect even statistically weak effects. All our findings have been replicated in random subsets of TARCC’s data. We have replicated all but one of our previously observed GDS-adjusted biomarker associations with δ, even though 1) our sample size has increased over time, 2) we are using a new δ homolog, 3) the biomarkers are being used to predict future cognitive performance, and 4) the prior associations were obtained using raw biomarker data while these employ normalized variables.

We have also independently confirmed 4/9 serum proteins previously recognized as depression-specific serum protein biomarkers by Papakostas et al. [44] and Bilello et al. [45]. We have additionally clarified that of those four, only A1AT and resistin are mediators of depressive symptoms’ dementing effect on disabling (i.e., “dementing”) cognitive performance.

We could not confirm Papakostas et al.’s association between five serum proteins and depression. [44] These included APOCIII, BDNF, Cortisol, EGF, and PRL. Each were associated with δ, but not the GDS. Additionally, BDNF and cortisol were associated with δ, but not the GDS. These have extensive depression-related literatures, and so this was also surprising.

However, our sample frame is ethnically diverse and has been selected against cases of clinical major depression. The mean GDS score is within the normal range and <20% of cases exhibited clinically significant GDS scores. Papakostas et al.’s [44] sample was much smaller, younger, had active major depressive episodes and histories of recurrent diatheses. Moreover, we could find no studies associating serum BDNF or cortisol specifically with the GDS in older persons. On the contrary, two studies failed to associate serum cortisol [47] or BDNF [48] with the GDS 15 and 30 respectively. Moreover, our results do confirm BDNF’s [52] and cortisol’s [53] reported associations with dementia severity, and we can also confirm both testosterone’s lack of an association with the GDS30 [54] and its significant association with dementia. [55]

We have identified four classes of proteins: 1) potential mediators of the GDS’s significant direct effect on δ, 2) δ-independent predictors of GDS, 3) GDS-independent predictors of δ, and 4) proteins unrelated to either the GDS or δ. While many proteins were related to the GDS, only a subset were associated with δ. δ in turn, has been associated with atrophy in the Default Mode Network (DMN)[56], as has dDEP” (i.e., a δ ortholog targeting the GDS).[57] This suggests that the mediators in Table 2 may be associated with the structure and /or function of the DMN. Thompson et al. [58] found that elevated serum protein S100B levels to be significantly correlated with DMN activity in older persons. S100B has been recognized as a potential treatment target in mood disorders [46] and is confirmed as a mediator of depressive symptoms’ disabling cognitive effects by this analysis. It is also interesting that all of the mediators in Table 2 have been previously associated with δ *in GDS adjusted models* [17–18]. This hints at depression independent effects of the same proteins on dementia-severity. For instance, IGF-1, FAS, resistin and S100B also mediate age’s effect on δ [17].

Our observations help clarify depression’s effects on cognitive function. First, although depressive symptoms may have both direct and indirect effects on observed cognitive performance, only indirect effects mediated by δ should be functionally salient, and thus “dementing.” This constrains the “pseudodementia” of depression to an effect on intelligence. This may limit the ability of antidepressant interventions to reverse depression’s more disabling (i.e., “dementing”) cognitive sequelae. Successful interventions on the “dementia of depression” (DoD) will have to effect changes in general intelligence, regardless of their effect on self-reported mood. This may explain the relatively slow trajectory of functional recovery after otherwise “successful” antidepressant treatment. [59–60] Unfortunately, we do not have access to antidepressant treatment data, and so we cannot test for associations between antidepressant exposure and δ.

One limitation to our study is that we tested univariate associations. We cannot address whether subsets of these proteins may be acting in concert to effect changes in dementia severity, nor do our results indicate the biological networks or systems through which they act. Such issues are beyond the scope of this data. However, it is unlikely that they contribute to a single dementing process. The wide range of serum proteins in Tables 2 and 3 suggest that they impinge upon the d-score through multiple processes. A further limitation is that these proteins were not rationally selected and TARCC’s panel, although extensive, is not exhaustive. However, our results have been Bonferroni corrected to reduce Type II error.

Nevertheless, the mediators identified in Table 2 may offer targets for the specific remediation of depression’s dementing cognitive effects. Several of these proteins may be embedded in networks that are amenable to pharmacological modulation. Alternatively, any individual protein might be modulated by a wide range of biological techniques.

Finally, although the GDS was weakly associated with prospective dEQ scores, even small changes in that construct are likely to be clinically meaningful [15]. The GDS is associated with a two-fold increased 5yr dementia conversion risk in TARCC. We have shown resistin, strongest of the Class 1 biomarkers (Table 2), to fully mediate that effect [61]. Resistin is reported to be elevated in AD cases [62]. Successful antidepressant treatment has been reported to reduce resistin levels [63]. This suggests some potential to modify δ scores in the context of antidepressant treatment. Because δ is agnostic to dementia etiology [11, 64], antidepressant intervention might improve a wide range of conditions, even in the presence of subsyndromal depressive symptoms.
